# Temporomandibular Joint Space Dimensions among Saudi Patients with Temporomandibular Disorders: MRI-Based Retrospective Study

**DOI:** 10.1155/2022/5846255

**Published:** 2022-08-02

**Authors:** Nasser Raqe Alqhtani, Malak Sultan Alkhaldi, Alhanoof Falah Alanazi, Abdullatif Saad Alabdulsalam, Adel Alenazi, Mahmud Uz Zaman, Adel Alzahrani, Ahmad Alshadwi, Ali Al Rafedah, Mohammed AlOtaibi

**Affiliations:** ^1^Department of Oral and Maxillofacial Surgery and Diagnostic Sciences, College of Dentistry, Prince Sattam Bin Abdullaziz University, Ad Dilam Rd, Ar Rashidiyah, Al-Kharj 16245, Saudi Arabia; ^2^Ministry of Defense, General Dentist, Armed Forces Hospital Southern Region, P.O. Box 101, 61961 Khamis Mushayt, Saudi Arabia; ^3^Alhabib Medical Group, General Dentist, Dr. Sulaiman Alhabib Hospital, P.O. Box 5612, Hamza Ibn Abdul Mutalib Street, Alsuwaidi AlGarbi, Riyadh 12994, Saudi Arabia; ^4^Ministry of Health, General Dentist, 2604 Muflah Street Al Shifa, Riyadh 14713, Saudi Arabia; ^5^Department of Oral Medicine and Diagnostic Sciences, College of Dentistry, King Saud University, PO Box 60169, Riyadh 11545, Saudi Arabia; ^6^Consultant of Oral and Maxillofacial Surgery, John Hopkins Aramco Health Care Services, 8131 Medical Access Rd No. 1, Gharb Al Dhahran, Dhahran 34465, Saudi Arabia; ^7^Consultant-Oral and Maxillofacial Surgery Department, Prince Sultan Military Medical City, As Sulimaniyah, Riyadh 12233, Saudi Arabia

## Abstract

**Introduction:**

The temporomandibular joint is a complex synovial joint in the body. It is the area in which the mandible articulates with the cranium. The temporomandibular joint space is located between the articular eminence and the glenoid fossa of the temporal bone at the base of the skull and the condylar process of the mandible. This interarticular space is divided into superior joint space (1.2 ml) and inferior joint space (0.9 ml) by the articular disc. The purpose of this study is to detect and evaluate the variations in the temporomandibular joint space among patients having temporomandibular joint disorders.

**Materials and Methods:**

In this retrospective study, 60 magnetic resonance imaging scans were evaluated at King Faisal Specialist Hospital in Riyadh, Saudi Arabia, between the years 2006 and 2016. Measurements were done in sagittal view in three areas: anterior, central, and posterior areas. However, coronal view readings were recorded in two different areas: medial and lateral joint spaces. All measurements were recorded at the highest point of the condyle that is perpendicular to the opposing bone. The SPSS program was used for statistical analysis.

**Results:**

The central joint space values were higher than the anterior and posterior joint spaces in both coronal and sagittal views. We also found that joint spaces among male patients were higher than female patients (right side *P*=0.015 and left side *P*=0.006). It is worth mentioning that the number of temporomandibular joint disorder female patients was more than the number of male temporomandibular joint disorder patients (52 females versus 24 males). Additionally, patients who were older than 55 years old had wider joint spaces than patients who were younger than 25 years old.

**Conclusion:**

The central joint space value was the highest among the other joint spaces on both views of magnetic resonance imaging, and the values of joint spaces among males were larger than those of females on sagittal magnetic resonance imaging. Patients with elderly temporomandibular joint disorders showed larger joint spaces than young patients. This study spotlights the importance of magnetic resonance imaging evaluation in temporomandibular joint disorder patients for a better understanding of the clinical evolution of temporomandibular disorders.

## 1. Introduction

The temporomandibular joint (TMJ) is one of the most fascinating and complex synovial joints in the body. It is the area in which the mandible articulates with the cranium [1]. The mandibular condyle is one part of the TMJ's bony components and is the portion of the mandible that connects it to the skull base. The condylar head anteroposterior dimension is found to be 8–10 mm and the mediolateral dimension is about 18–23 mm [1, 2]. The articular disc (AD) is composed of fibrocartilage and crimped type I collagen, which is thought to be better at absorbing impacts. The AD morphology is biconcave in the sagittal section, divided into anterior and posterior bands, and the middle part is between the two, which is the thinnest part of the AD [3, 4].

There are two joint spaces which are separated by the AD; the superior and inferior joint spaces separate the glenoid and articular eminence of the temporal bone into two components and are positioned between the condylar head and glenoid fossa/articular eminence during the rotation and translation motion of the mandible [5].

The anterior transformation occurs during opening of the mouth between the discs and the mandibular articular eminence of the temporal bone [6]. The structural changes in the TMJ, including bony and discal measurement, are almost related to the aetiology of TMJ disorders (TMD) [7–9].

Marques AP et al. showed a high level of sensitivity and specificity using cone beam computed tomography (CBCT) to identify mandible condyle lesions. But, in this study, we focused on determining the discal space relation with the condylar head without any osteoarthritic changes [10]. Nowadays, magnetic resonance imaging (MRI) is widely used to determine the soft tissue pathology of the TMJ. Petersson suggested using T1 or proton density sequences in combination with T2 images in the case of TMD. In addition, he also mentioned using the MRI machines that have field strengths of 1.5 Tesla and axial localizers to correct the direction of the slice to obtain slices perpendicular to the condylar axis in sagittal views and parallel to the condylar axis in coronal views [11].

In this study, we investigated the relationship between changes in the discal space and patients having TMD. The link has not yet been clarified. To date, there have been no studies investigating this relationship among the Saudi population; therefore, we conducted this study to detect and check if there is a link between the volumetric changes in the TMJ space and disc thickness among TMD patients in Saudi Arabia.

## 2. Materials and Methods

This study was approved by the Institutional Review Board (IRB) of King Faisal Specialist Hospital and Research Centre, Riyadh, Saudi Arabia. In this retrospective study, TMJ MRIs were collected from King Faisal Specialist Hospital. The medical records of patients who were referred to the Department of Oral and Maxillofacial Surgery with temporomandibular joint disorders were reviewed between the years 2006 and 2016. Patients with TMD who underwent MRI for diagnostic purposes were included in this study. Patients with a history of any systemic disease that will cause structural or physiological changes to the TMJ and surrounding structure (e.g., osteoporosis, systemic lupus erythematosus, idiopathic juvenile arthritis, and rheumatoid arthritis) were excluded from the study, as were patients with head and neck neoplasms or TMJ surgery. The study included 76 patients (152 TMJs); however, 16 cases were excluded due to poor image quality and condylar atrophy. The final sample of 60 cases was analysed. We assessed each MRI scan's DICOM files using RadiAnt DICOM viewer version 4.5.9 (Medixant, Poznan, Poland). Images were viewed on a 27-inch Dell UltraSharp LCD monitor with a resolution of 3840 × 2160 pixels (Dell Technologies, Round Rock, Texas, U.S.). The patients' MRIs were examined, and all measurements and analyses were performed by two dental surgeons, who were trained by an experienced oral and maxillofacial radiologist. Crombach's alpha value was 1 (>0.9) when all inter and intraexaminer reliabilities were calculated with the ICC intraclass correlation coefficient. This indicates that the inter and intraexaminer reliabilities between the two examiners at the baseline and after 15 days were excellent.

**Figure 1:**
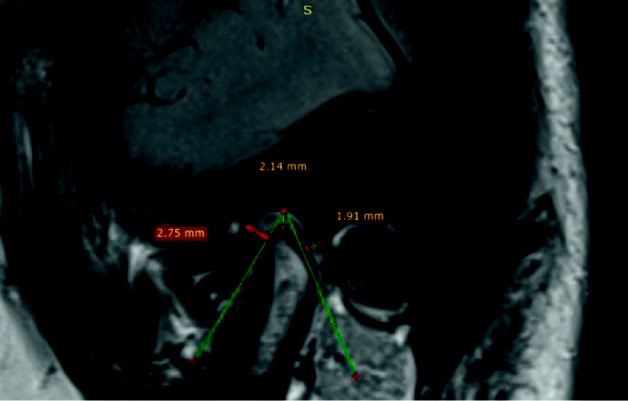
Measuring the TMJ space in MRI sagittal view (anterior joint space = 2.75 mm, central joint space = 2.14 mm, and posterior joint space = 1.91 mm).

MRI scans were obtained using a 3-T imager with a head coil (MAGNETOM Trio, Siemens Healthineers USA). The patients were imaged using a proton density (PD)-weighted spin-echo (SE) MRI sequence in the closed mouth position (TR/TE 2400/20; 2 mm slice thickness for both sagittal and coronal views; 150 mm FOV; 256 × 256 matrices). The analysis of each MRI was done from two views (sagittal and coronal) in a closed mouth position. We measured three sites in the sagittal view (anterior joint space, central joint space, and posterior joint space). In this image, for example, the central joint space = 2.14 mm (Figure 1), so we drew a line from the fossa's centre and dragged it until it touched the posterior border of the condyle; then, we drew a perpendicular line to the point where it touches the posterior surface of the condyle and it has to be a 90° angle to the line, and we recorded it (e.g., in this MRI, the way with the anterior joint space, we drew a line from the centre of the mandibular fossa and dragged it until it touched the anterior border of the condyle. Then, we drew a perpendicular line to the point where it touches the anterior surface of the condyle. It has to be a 90° to the line, and then, we recorded it (e.g., MRI anterior joint space = 2.75 mm (Figure 1)).

**Figure 2:**
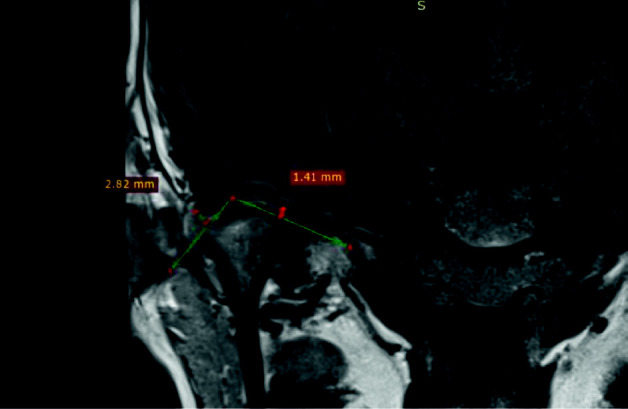
Measuring the TMJ space in MRI coronal view (medial pole = 1.41 mm and lateral pole = 2.82 mm).

The other two sites were measured in the coronal view (medial and lateral). The measurements were done on the medial pole and the lateral pole by drawing a line from the middle of the mandibular fossa and dragging it obliquely until it is touching the medial pole and then drawing another line that is perpendicular to the first line to measure the space (e.g., medial pole = 1.41 mm and lateral pole = 2.82 mm (Figure 2).

Both sagittal and coronal views were evaluated in the closed mouth position. Open mouth position is not measured in TMD patients because of condylar discrepancies, anterior disc displacements, and the limitation of mouth opening in some patients.

All data analyses were accomplished with the SPSS program (version 20 for Windows, IBM Corp.). Descriptive statistics like mean and standard deviations were tabulated. Statistically significant differences between the male and female groups were assessed by the independent sample *t*-tests and between different age groups were analysed with one-way ANOVA by keeping the significance level at *P*0.05.

## 3. Results

**Figure 3:**
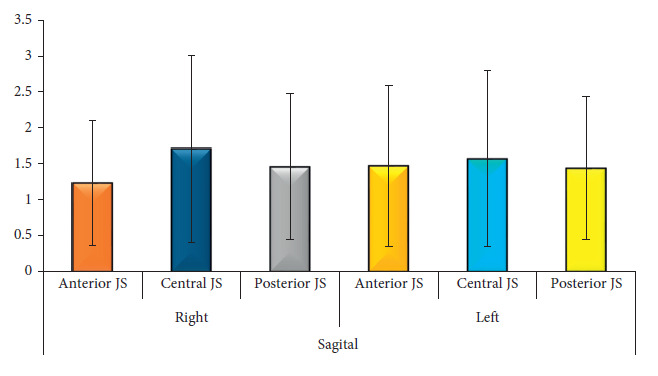
The mean distribution of MRI readings of a sagittal view of the left and right TMJ.

**Figure 4:**
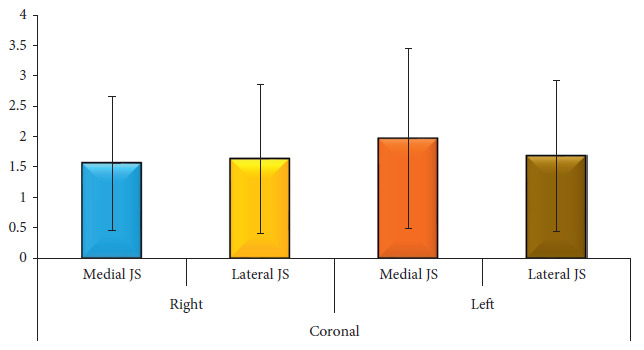
The mean distribution of MRI readings of a coronal view of the left and right TMJ.

The mean anterior sagittal (AS), central sagittal (CS), and posterior sagittal (PS) values of the right side were 1.22 mm (SD 0.869 mm), 1.7 mm (SD 1.3 mm), and 1.46 mm (SD 1.01 mm), respectively. The ratio of AS:CS:PS was 1.0:1.4:1.2. Similarly, the values on the left side were 1.47 mm (SD 1.12 mm), 1.56 mm (SD 1.22 mm), and 1.44 mm (SD 0.998 mm), with the ratio of AS:CS:PS being 1.0:1.06:0.98. Whereas, the coronal view via MRI demonstrated the medial and lateral measurement of disc space. The mean coronal medial (CM) joint space measurement in MRI of the right condyle was 1.56 mm (SD 1.1 mm) and the lateral joint space measurement was 1.63 mm (SD 1.23 mm) and the ratio of CM:CL was 1.0:1.04. Similarly, on the left side, coronal medial joint space was found to be 1.97 mm (SD 1.5 mm) and coronal lateral joint space was 1.69 (SD 1.23 mm), with the ratio of CM:CL being 1.0:0.86 (Table 1, Figures 3 and 4).

**Figure 5:**
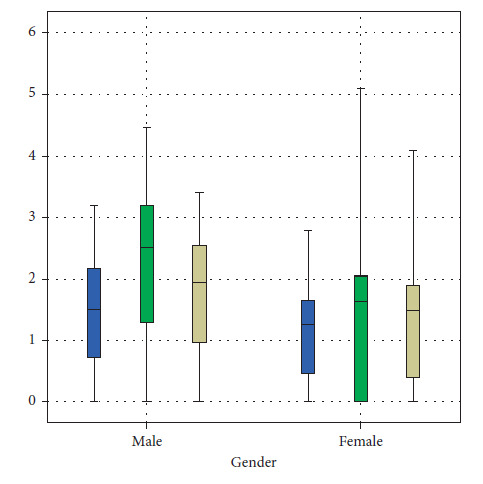
Distribution of MRI readings of the sagittal view of the right TMJ between males and females.

**Figure 6:**
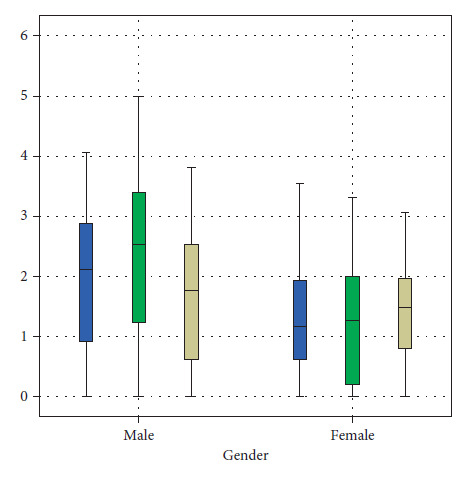
Distribution of MRI readings of the sagittal view of the left TMJ between males and females.

**Figure 7:**
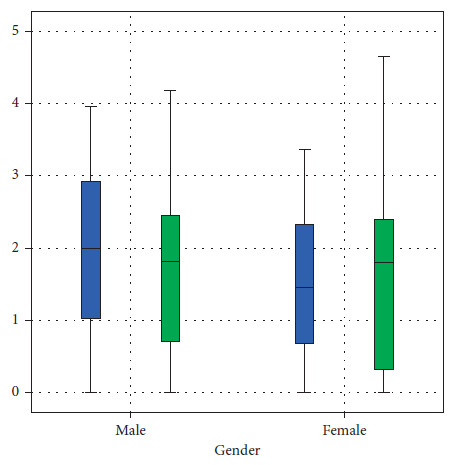
Distribution of MRI readings of the coronal view of the right TMJ between males and females.

**Figure 8:**
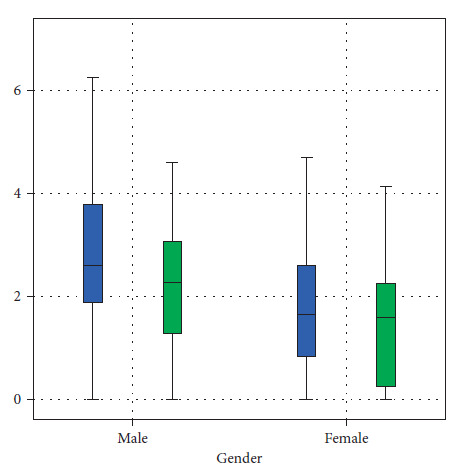
Distribution of MRI readings of the coronal view of the left TMJ between males and females.

The mean sagittal right central JS values display a statistically significant higher value (*P*=0.015) among males (2.231.4) when compared with the female values (1.461.18). Similarly, sagittal left anterior JS (*P*=0.034), central JS (*P*=0.006), and medial left coronal values displayed a statistically significant higher value among males when compared to females (Table 2, Figures 5–8).

Similarly, when a comparison of MRI readings of sagittal and coronal views of the left and right TMJ was performed between different age groups and on different sides (left and right), it exhibited no statistical difference (*P* > 0.05) (Table 3).

Similarly, MRI readings of the right-sided anterior (*P*=0.849), central (*P*=0.351), and posterior JS (*P*=0.21) of TMJ and MRI readings of left-sided anterior (*P*=0.91), central (*P*=0.429), and posterior JS (*P*=0.41) of TMJ in sagittal view exhibited no statistically significant change between different age groups.

The MRI readings of the right side medial (*P*=0.544) and lateral JS (*P*=0.089) of TMJ and MRI readings of the left side medial (*P*=0.302) and lateral JS (*P*=0.316) of TMJ in coronal view revealed no statistically significant alteration between different age groups.

## 4. Discussion

TMJ imaging is regarded as one of the challenging areas to be gauged by routine radiographs (panoramic radiography) and conventional plain radiographs (TMJ projections) because of the superimposition of neighbouring structures and its low sensitivity and precision for deeper structures [11, 12]. The morphology and three-dimensional relationships of the condyle and the fossa have been studied with radiographic techniques that include conventional tomography [13–15] and computed tomography (CT) [16, 17]. Magnetic resonance imaging is unique in evaluating cartilage degeneration, loss, disc displacement, bony changes, and soft tissue variations such as inflammatory synovial proliferation [13, 18–20]. Bony changes and inflammation will have a direct influence on the joint space. Hence, MRI would be one of the most efficient and reliable choices for TMJ space analysis. The measurements of the joint space were introduced by Ricketts [21, 22] to describe the eccentric or concentric condylar positions on transcranial radiographs. The measurements of the anterior and posterior joint space were performed to distinguish patients with temporomandibular disorders (TMDs) from asymptomatic controls, considering the symmetrical position of the condyle as an indicator of normal [23, 24]. Only a few studies focused exclusively on joint space measurement with TMD. The joint space is loosely connected with the aetiology of TMJ disorders (TMD); awareness of the morphological physiognomies of the TMJ is of greatest importance to the clinician [25–27]. The clinical significance of joint space is of great value. The widening or obliteration of the joint space may correlate with TMJ disease and its pathology [25–27]. The disc space has been speculated to impact the condylar position of the contralateral joint [21]. There is a discrepancy in TMJ space, which assists in diagnosing the TMD with unilateral or bilateral disk displacements [5].

The sagittal and coronal views of TMJ by MRI can give a clearer picture of the joint space of TMJ for analysis. The anterior joint space, central joint space, and posterior joint space can be measured on sagittal images of TMJ [25, 28], and the medial and lateral joint space can be measured on a coronal view [25]. In the present study, we adopted the combination of sagittal and coronal views in the determination of the joint space measurement, and to rule out additional surrounding pathology, closed mouth coronal and sagittal sequences were incorporated over an open mouth to analyse the general anatomy, space, and bone marrow, as well as the adjacent soft tissues [5]. There is a difficulty in having a standardized measurement in the open mouth position because of different temporomandibular joint disorders that result in anterior disc displacement and condylar position discrepancies.

In this study, the number of female subjects with TMD is higher than that of their male counterparts, which is in parallel with the systematic review on the prevalence of TMD in the general population [29]. Whereas, in the present study, the prevalence of TMD is peaking in older subjects above 55 years, which is in disagreement with several cross-sectional studies showing the subject's age range of 20–40 years [29–31].

The sagittal central joint space on both sides, sagittal superior joint space on the right side, and coronal medial joint space on the left side exhibited statistically significant differences between the sexes. This indicates the sagittal central joint space of both sides, the superior joint space of the sagittal view of the right side, and the medial joint space of the coronal view on the left side wer larger among males than females. This finding is in parallel with the results of Kinniburgh et al.' [32] study and disagrees with the results of the study conducted by Hansson et al. [33] who observed no significant difference in the values of the joint space between the sexes. In the present study, different age groups did not have any influence on the joint space, which is similar to the results from the systematic review by Panchbhai [25]. The difference in the results of different studies may be associated with the social, demographic, cultural, and lifestyle patterns of the studied population.

The present study demonstrated no significance when comparing the joint spaces between the right and left sides, which is in agreement with most of the published studies [25, 34, 35]. However, some studies were contradictory to our findings; they showed significant differences for the anterior and posterior joint space [25, 36–38]. This asymmetry could be related to atypical cranial base or mastication side preferences [37]. Furthermore, most patients have an acentric relation-centric occlusion discrepancy, which is frequently caused by unilateral posterior interference. To illustrate, the condyles might shift asymmetrically, while the contralateral condyle moves sagittally, and the ipsilateral rotates to have a more balanced dental occlusion [37].

The present study possesses some generalised limitations like other research and some of the biological factors such as age, sex, and physiognomy can contribute to the residual disparity of condylar position and disc space. Besides, the time factor that runs between the incidence of disk movements and MRI-based measurements might have contributed to the disparity because this time span was not the same for person to person. Thus, adaptive processes can also be a discrepancy source.

Overall, there were more differences in the study findings with respect to joint space analysis, comparisons, and association with other parameters. Furthermore, larger prospective studies are required in this field to generalise the findings.

## 5. Conclusion

This study showed that the central joint space values were higher than the anterior and posterior joint spaces in both coronal and sagittal views among the Saudi population. We also found that joint spaces among male patients were higher than in female patients. It is worth mentioning that the number of TMD female patients was more than the number of male TMD patients (52 females versus 24 males). Additionally, patients who were older than 55 years old had wider joint space than patients who were younger than 25 years old.

## Figures and Tables

**Table 1:** The mean and standard deviation of MRI readings of the sagittal and coronal view of the left and right TMJ.

			*N*	Mean	Std. deviation
Sagittal	Right	Anterior JS	76	1.231	0.869
		Central JS	76	1.703	1.298
		Posterior JS	76	1.457	1.016
	Left	Anterior JS	76	1.470	1.120
		Central JS	76	1.566	1.229
		Posterior JS	76	1.438	0.998

Coronal	Right	Medial JS	76	1.563	1.104
		Lateral JS	76	1.634	1.230
	Left	Medial JS	76	1.968	1.477
		Lateral JS	76	1.681	1.238

**Table 2:** Comparison of MRI readings of sagittal and coronal views of the left and right TMJ between males and females.

			Gender	*N*	Mean	SD	Std. error mean	*t* value	*P* value
Sagittal	Right	Anterior JS	Male	24	1.40	0.97	0.20	1.169	0.246
			Female	52	1.15	0.82	0.11		
		Central JS	Male	24	2.23	1.40	0.29	2.497	0.015^*∗*^
			Female	52	1.46	1.18	0.16		
		Posterior JS	Male	24	1.75	1.07	0.22	1.725	0.089
			Female	52	1.32	0.97	0.13		
	Left	Anterior JS	Male	24	1.93	1.35	0.28	2.217	0.034^*∗*^
			Female	52	1.26	0.93	0.13		
		Central JS	Male	24	2.24	1.50	0.31	2.974	0.006^*∗*^
			Female	52	1.25	0.95	0.13		
		Posterior JS	Male	24	1.66	1.17	0.24	1.35	0.181
			Female	52	1.33	0.90	0.13		

Coronal	Right	Medial JS	Male	24	1.90	1.24	0.25	1.848	0.069
			Female	52	1.41	1.01	0.14		
		Lateral JS	Male	24	1.70	1.30	0.26	0.336	0.738
			Female	52	1.60	1.21	0.17		
	Left	Medial JS	Male	24	2.61	1.75	0.36	2.655	0.01^*∗*^
			Female	52	1.67	1.24	0.17		
		Lateral JS	Male	24	2.10	1.47	0.30	1.818	0.078
			Female	52	1.49	1.08	0.15		

^*∗*^Statistical significance set at 0.05.

**Table 3:** Comparison of the MRI readings of the sagittal and coronal views of the left and right TMJ between different age groups.

				*N*	Mean	SD	*P* value
Sagittal	Right	Anterior JS	Less than 25	11	1.02	0.98	0.849
			25–34	37	1.24	0.87	
			35–44	13	1.35	0.81	
			45–54	11	1.17	0.96	
			55 and above	4	1.53	0.76	
		Central JS	Less than 25	11	1.12	0.95	0.351
			25–34	37	1.70	1.28	
			35–44	13	1.86	1.08	
			45–54	11	1.79	1.51	
			55 and above	4	2.62	2.16	
		Posterior JS	Less than 25	11	1.24	1.17	0.21
			25–34	37	1.38	0.90	
			35–44	13	1.64	0.98	
			45–54	11	1.32	1.20	
			55 and above	4	2.54	0.92	
	Left	Anterior JS	Less than 25	11	1.37	1.29	0.91
			25–34	37	1.49	1.15	
			35–44	13	1.54	1.12	
			45–54	11	1.28	1.06	
			55 and above	4	1.89	0.86	
		Central JS	Less than 25	11	1.28	1.34	0.429
			25–34	37	1.56	1.11	
			35–44	13	1.45	1.06	
			45–54	11	1.61	1.65	
			55 and above	4	2.66	1.16	
		Posterior JS	Less than 25	11	1.31	1.16	0.441
			25–34	37	1.53	0.99	
			35–44	13	1.41	1.08	
			45–54	11	1.05	0.81	
			55 and above	4	2.08	0.79	
Coronal	Right	Medial JS	Less than 25	11	1.11	1.05	0.544
			25–34	37	1.68	1.06	
			35–44	13	1.57	1.13	
			45–54	11	1.45	1.19	
			55 and above	4	2.05	1.46	
		Lateral JS	Less than 25	11	1.00	0.95	0.089
			25–34	37	1.75	1.23	
			35–44	13	1.80	1.42	
			45–54	11	1.26	1.04	
			55 and above	4	2.77	0.95	
	Left	Medial JS	Less than 25	11	1.27	1.25	0.302
			25–34	37	2.06	1.50	
			35–44	13	1.90	1.42	
			45–54	11	2.04	1.65	
			55 and above	4	3.07	1.31	
		Lateral JS	Less than 25	11	1.49	1.46	0.316
			25–34	37	1.59	1.15	
			35–44	13	1.89	1.29	
			45–54	11	1.49	1.18	
			55 and above	4	2.87	1.33	

^*∗*^Statistical significance set at 0.05.

## Data Availability

The data used to support the findings of this study are available from the corresponding author upon request.
